# Trends and Outcomes in Patients With Coronary Artery Disease Undergoing TAVR: Insights From VA CART

**DOI:** 10.1016/j.jscai.2023.101056

**Published:** 2023-06-27

**Authors:** Khanjan B. Shah, Colin O’Donnell, Dhruv Mahtta, Stephen W. Waldo, Calvin Choi, Ki Park, Ali E. Denktas, David Paniagua, Umair Khalid

**Affiliations:** a Division of Cardiovascular Medicine, University of Florida, Gainesville, Florida; b Malcolm Randall VA Medical Center, Gainesville, Florida; c VA Clinical Assessment, Reporting and Tracking (CART) Program, VHA Office of Quality and Patient Safety, Washington, DC; d Section of Cardiology, Department of Medicine, Baylor College of Medicine, Houston, Texas; e Rocky Mountain Regional VA Medical Center, Aurora, Colorado; f University of Colorado School of Medicine, Aurora, Colorado; g Section of Cardiology, Medical Care Line, Michael E. DeBakey VA Medical Center, Houston, Texas

**Keywords:** Clinical Assessment, Reporting and Tracking Program, percutaneous coronary intervention, transcathter aortic valve replacement, Veterans Affairs

## Abstract

**Background:**

Obstructive coronary artery disease (CAD) is common in patients with severe symptomatic aortic stenosis. The management and impact of obstructive CAD in patients undergoing transcatheter aortic valve replacement (TAVR) have not been fully evaluated. We aimed to determine the patient characteristics and clinical outcomes among veterans undergoing TAVR with and without obstructive CAD and to determine temporal trends and association of pre-TAVR percutaneous coronary intervention (PCI) with clinical outcomes.

**Methods:**

We identified all patients who underwent TAVR from 2012 to 2021 in the VA Health Care System. The sample population was divided into patients with and without obstructive CAD and further stratified by coronary intervention status 1 year prior to TAVR. The primary outcome was 1-year all-cause mortality, and the secondary outcome was major bleeding.

**Results:**

During the study period, 759 patients underwent TAVR, and 282 (37%) had obstructive CAD. Obstructive CAD was associated with higher 1-year mortality (15.6% vs 7.1%; *P* < .01) after TAVR. The rate of PCI prior to TAVR increased from 2012 until 2016, after which it steadily declined such that 144 patients (51%) underwent PCI pre-TAVR during the entire study period. There was no difference in 1-year mortality (16.0% vs 15.2%; *P* = .89) or bleeding (16.7% vs 12.3%; *P* = .33) between patients who underwent or did not undergo pre-TAVR PCI.

**Conclusions:**

Among veterans undergoing TAVR, the presence of obstructive CAD is associated with higher mortality though pre-TAVR coronary intervention is not associated with improved outcomes. Further studies could identify a subset of patients who may benefit from coronary revascularization prior to TAVR.

## Highlights

• Patients with obstructive coronary artery disease undergoing transcatheter aortic valve replacement have worse 1-year survival compared with those without obstructive coronary artery disease.
• Percutaneous coronary intervention prior to transcatheter aortic valve replacement did not improve 1-year outcomes among patients undergoing transcatheter aortic valve replacement in the Veterans Affairs system.
• Percutaneous coronary intervention prior to transcatheter aortic valve replacement decreased over the study period suggesting a trend toward a more conservative strategy for managing obstructive coronary artery disease.

## Central Illustration

**Figure:**
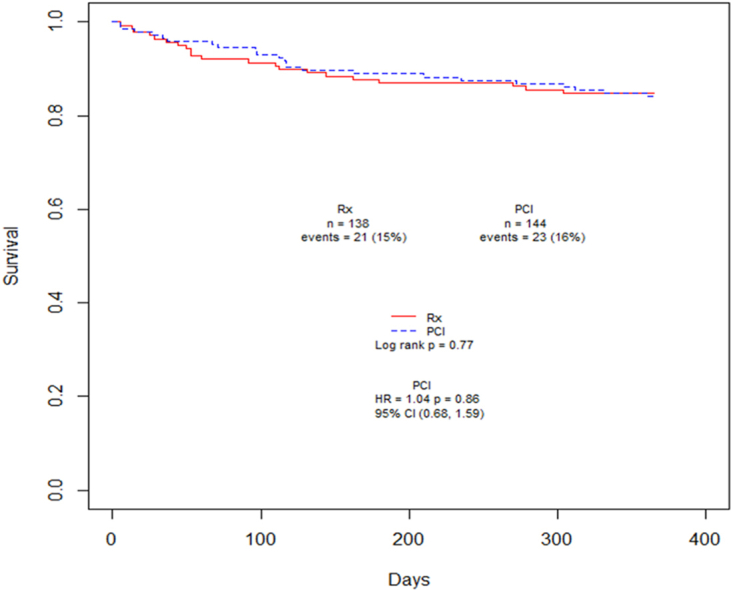

## Introduction

Severe aortic stenosis is the most common valvular heart disease of the elderly, with an estimated prevalence of 2% to 4% in patients >75 years.^1^ Due to a preponderance of high quality evidence, transcatheter aortic valve replacement (TAVR) is now the predominant form of aortic valve replacement in the United States.^2^^,^^3^

Obstructive coronary artery disease (CAD) is estimated to be present in 40% to 60% of patients with severe symptomatic aortic stenosis undergoing valve replacement.^4^ Despite the high prevalence of concomitant obstructive CAD and severe symptomatic aortic stenosis, data are limited regarding the impact of obstructive CAD and subsequent revascularization on outcomes following aortic valve replacement. A recent observational study demonstrated higher mortality in patients with obstructive CAD with no difference in mortality after pre-TAVR percutaneous coronary intervention (PCI). Importantly, this study was limited to a single-center experience with a homogenous patient population.^5^ Evaluation in a large national cohort has not yet been performed.

With this in mind, we sought to determine the association of obstructive CAD on clinical outcomes among patients undergoing TAVR. Furthermore, we sought to determine temporal trends and association of pre-TAVR PCI with clinical outcomes in the same population.

## Methods

### Study population and data source

**Figure 1:**
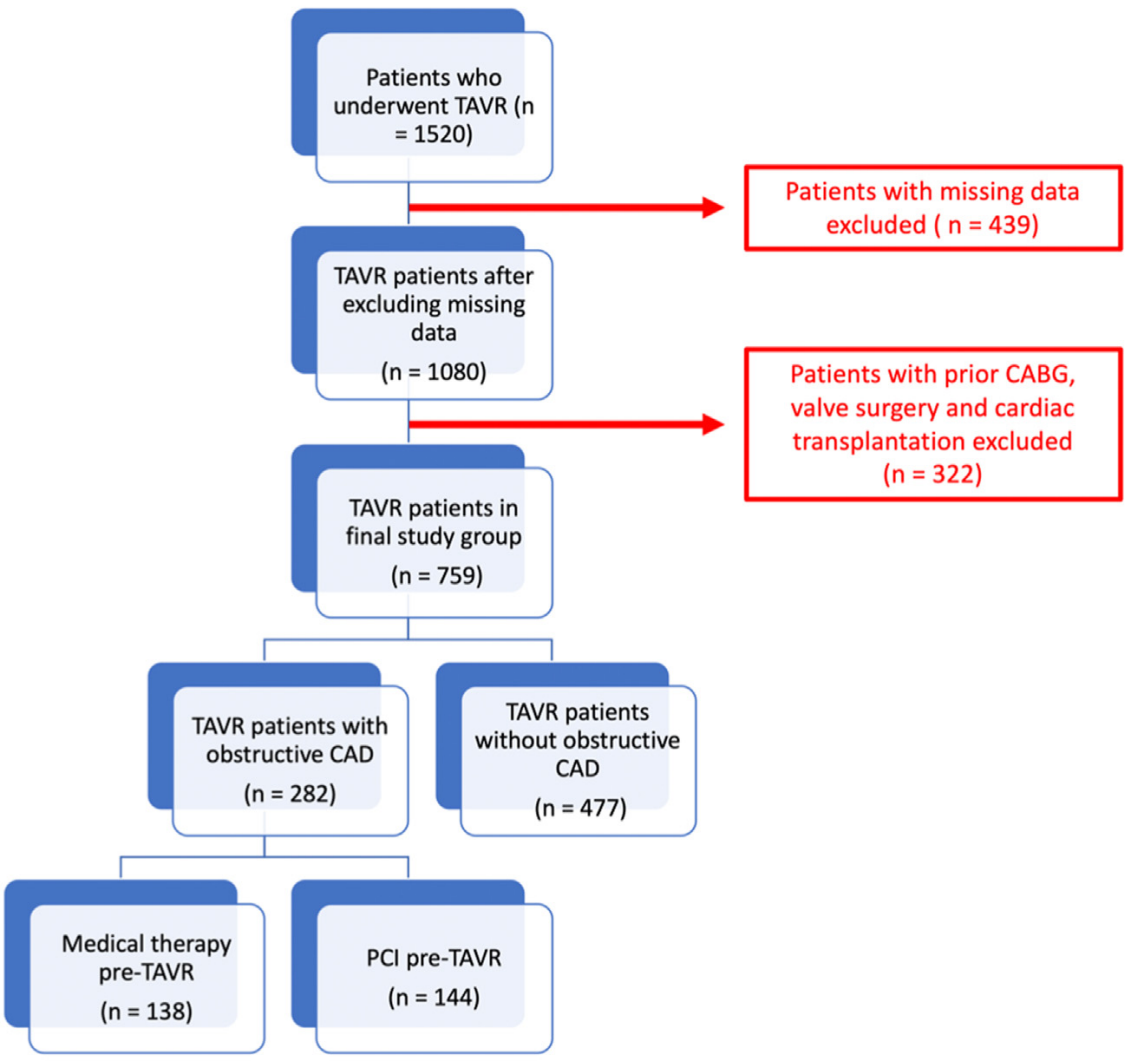
**Flowchart showing study population after exclusion criteria.** Patients are further stratified by the presence of absence of obstructive CAD and pre-TAVR medical therapy or PCI among patients with obstructive CAD. CAD, coronary artery disease; PCI, percutaneous coronary intervention; TAVR, transcatheter aortic valve replacement.

The Veterans Affairs (VA) Clinical Assessment, Reporting and Tracking (CART) Program is a national quality and safety organization for several medical disciplines within the VA Health Care System. As part of this mission, the CART Program collects clinical and procedural information on all patients undergoing invasive cardiac procedures throughout the enterprise. The present study queried this dataset to identify all patients who underwent TAVR at a VA facility from 2012 to 2021. There are a limited but growing number of TAVR sites within the VA system, all of which are required to report to CART by Veterans Health Administration directive.^6^ Patients with prior coronary artery bypass graft, valve surgery, cardiac transplantation, and missing anatomic information precluding the calculation of anatomic complexity with the VA SYNTAX score were excluded (Figure 1). After the above exclusions, the sample population was divided into patients with and without obstructive CAD, with obstructive CAD defined as a VA SYNTAX score >0.^7^ The VA SYNTAX score is a validated score that is automatically generated in the VA CART system using anatomic characteristics, such as location of stenosis, coronary dominance, and presence of calcification. Patients with CAD were further stratified by the performance of coronary intervention within 1 year prior to TAVR. The indication for PCI as compared to medical management of obstructive CAD was at the discretion of the operator and was not captured in our dataset. The study was deemed exempt from review by the Colorado Institutional Review Board given the retrospective, deidentified nature of the dataset.

### Outcome definitions

The primary outcome was 1-year all-cause mortality. Major bleeding at 1 year was deemed a secondary outcome, defined by administrative codes for bleeding and supplemented with a transfusion or a >2 g/dL decline in hemoglobin approximating Bleeding Academic Research Consortium 3a bleeding events. An additional secondary outcome was major adverse cardiac events (MACE), defined as cardiovascular events or death, myocardial infarction, or hospitalization for heart failure.

### Statistical analyses

Testing for dichotomous covariates used Barnard’s exact 2×2 test to obtain *P* values under the null hypothesis of no difference in the binomial proportions. Testing of continuous covariates used *t* tests and nonparametric Mann–Whitney *U* tests. A measure of the skewness of each covariate was reported to determine if the assumptions of the parametric *t* test was violated and that the nonparametric test was more appropriate. Standardized differences for differences in proportions and for continuous covariates used the accepted standard formulas.

To ascertain if there was a trend in the percentage of PCIs prior to TAVR across years, and if the trend had a change point, a loess algorithm was first used to smooth the data. Then PROC MCMC in SAS was used on the smoothed data points to determine if there was a change point in the trend and to find its location. The least squares regression had a term for a single change point, below which a fixed slope for trend would be calculated and above which the slope would have a different value. The MCMC algorithm requires a prior distribution for the change point, which was given as uniform over the years 2012 to 2021. There were 1000 burn-in iterations and 20,000 iterations used to locate the change point in trend.

A Kaplan–Meier graph (product limit method) was generated to visualize the event rate between the specific study arms of each graphic and to obtain a log-rank statistic to indicate whether there was a significant separation between study arms across the covariate of interest. The hazard ratios for the covariates of interest were determined using accelerated failure time models that incorporated a random term for hospital cluster effect.

All tests used a 0.05 type I error rate to determine significance. SAS version 9.04.01M6 and R version 4.2.1 were used for all statistical analyses.

## Results

### Population characteristics

A total of 1520 patients without prior cardiac surgery underwent TAVR within the VA Health Care System from 2012 to 2021. After excluding patients with incomplete information on preprocedural coronary anatomy, a total of 759 patients remained in our study group, and 282 (37.2%) patients had obstructive CAD. Of patients who had obstructive CAD, 144 (51.1%) were treated with PCI prior to TAVR (Figure 1).

Baseline characteristics of patients undergoing TAVR with obstructive CAD compared with those without obstructive CAD are summarized in Table 1. Patients with obstructive CAD were more likely to be older and have comorbid conditions of heart failure and peripheral arterial disease. As seen in Table 2, there was no difference between those who did or did not undergo PCI prior to TAVR.

**Table 1:** Baseline characteristics of patients undergoing TAVR with coronary artery disease compared with those without coronary artery disease

Variable	TAVR without CAD (n = 477)	TAVR with CAD (n = 282)	*P*	Standardized difference
Age, y	78.1	79.4	.03	0.11
Male sex, %	96.6	98.2	.24	0.10
Body mass index, kg/m^2^	29.2	28.8	.40	0.04
Diabetes mellitus, %	44.6	51.4	.08	0.14
Hypertension, %	91.4	93.3	.42	0.07
Hyperlipidemia, %	87.6	88.6	.72	0.03
Congestive heart failure, %	40.9	49.6	.02	0.18
Atrial fibrillation, %	31.2	34.8	.42	0.08
Chronic kidney disease, %	36.7	36.1	.90	0.01
Peripheral arterial disease, %	28	37.2	.01	0.20
Prior stroke or transient ischemic attack, %	13.2	16.3	.25	0.09
Obstructive sleep apnea, %	28.9	28.7	.98	<0.01
Chronic obstructive pulmonary disease, %	36.1	28.7	.04	0.16
Tobacco use, %	59.1	59.6	.98	0.01
Alcohol abuse, %	7.1	6.0	.58	0.04

CAD, coronary artery disease; TAVR, transcatheter aortic valve replacement.

**Table 2:** Demographic characteristics of patients with coronary artery disease, with or without percutaneous coronary intervention, who underwent transcatheter aortic valve replacement

Variable	Medical therapy pre-TAVR (n= 138)	PCI pre-TAVR (n = 144)	*P*	Standardized difference
Age, y	80.2	78.7	.12	0.13
Male sex, %	98.6	97.9	.77	0.05
Body mass index, kg/m^2^	28.6	29	.55	0.05
Diabetes, %	50.7	52.1	.85	0.03
Hypertension, %	90.6	95.8	.08	0.21
Hyperlipidemia, %	89.1	88.2	.83	0.03
Congestive heart failure, %	48.6	50.7	.78	0.04
Atrial fibrillation, %	34.8	34.7	>.99	<0.01
Chronic kidney disease, %	34.1	38.2	.53	0.09
Peripheral arterial disease, %	32.6	41.7	.13	0.19
Prior stroke or transient ischemic attack, %	14.5	18.1	.53	0.10
Obstructive sleep apnea, %	23.9	33.3	.09	0.21
Chronic obstructive pulmonary disease, %	28.3	29.2	.89	0.02
Tobacco use, %	57.2	61.8	.53	0.09
Alcohol abuse, %	5.1	6.9	.53	0.08

CAD, coronary artery disease; PCI, percutaneous coronary intervention; TAVR, transcatheter aortic valve replacement

### Procedural outcomes

**Figure 2:**
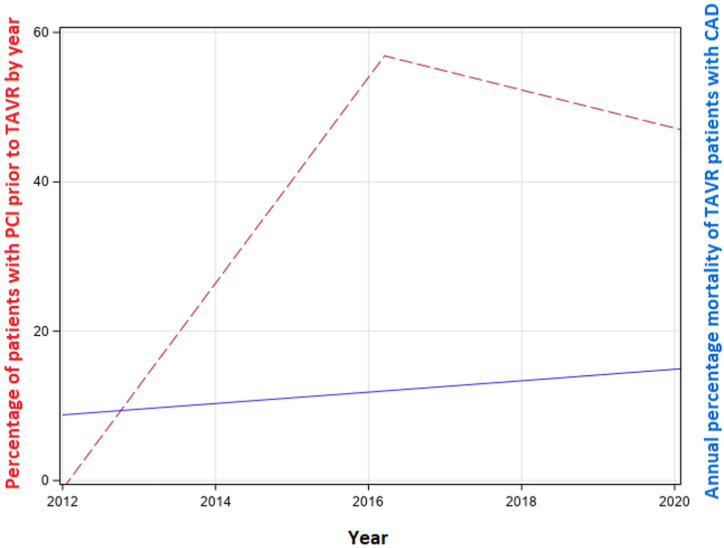
**Temporal trends in pre-TAVR PCI demonstrate that PCI increased from 2012 until 2016, after which PCI steadily declined**. Regardless, the overall annual mortality remained unchanged throughout the study period. PCI, percutaneous coronary intervention; TAVR, transcatheter aortic valve replacement.

Seventy-eight patients died within 1 year of TAVR (10.3%). Patients with CAD had significantly higher 1-year mortality compared with those without CAD (15.6% vs 7.1%; *P* < .01). However, the 1-year mortality did not differ whether these patients with obstructive CAD underwent PCI prior to TAVR (16.0% vs 15.2%; *P* = .89). The rate of PCI prior to TAVR increased from 2012 until 2016, after which it steadily declined (Figure 2).

A minority of patients (108, 14.2%) had major bleeding within 1 year of TAVR. The 1-year bleeding rates did not differ between those with coronary disease and those without (14.5% vs 14.0%; *P* = .86). As shown in Table 3, the 1-year bleeding also did not differ among those that did or did not undergo PCI prior to TAVR (16.7% vs 12.3%; *P* = .33).

**Table 3:** Clinical outcomes of patients who underwent TAVR, with and without CAD, with and without pre-TAVR PCI

Category of patients	1-year mortality		1-year major bleeding	
Patients undergoing TAVR	78/759 (10.3%)		108/759 (14.2%)	
With CAD (37%)	44/282 (15.6%)	*P* < .01	41/282 (14.5%)	*P* = .86
Without CAD (63%)	34/477 (7.1%)		67/477 (14.0%)	
Patients undergoing TAVR with CAD	44/282 (15.6%)		41/282 (14.5%)	
Pre-TAVR PCI (51%)	23/144 (16.0%)	*P* = .89	24/144 (16.7%)	*P* = .33
Pre-TAVR medically managed (49%)	21/138 (15.2%)		17/138 (12.3%)	

CAD, coronary artery disease; PCI, percutaneous coronary intervention; TAVR, transcatheter aortic valve replacement

**Figure 3:**
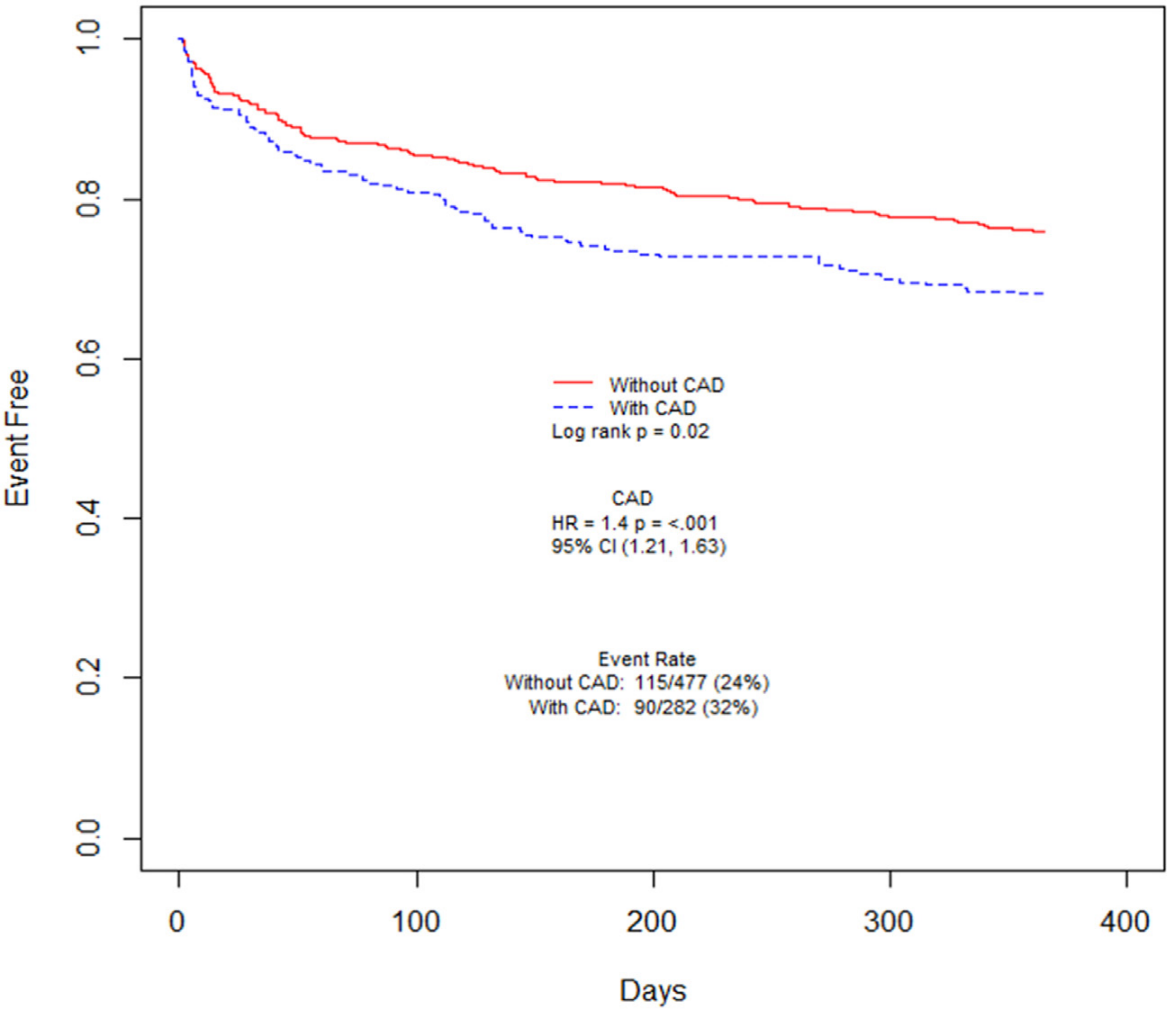
**Kaplan–Meier curves showing MACE-free rates in patients undergoing TAVR as stratified by obstructive CAD status**. Patients with obstructive CAD demonstrate lower MACE-free rates compared with patients without obstructive CAD. CAD, coronary artery disease; MACE, major adverse cardiac event; TAVR, transcatheter aortic valve replacement.

Kaplan–Meier figures were created for the study outcomes. Figure 3 shows MACE-free rates in patients who underwent TAVR with obstructive CAD compared with those without obstructive CAD. There was no difference in mortality rates over 1 year between those TAVR patients that did or did not undergo PCI prior to TAVR (Central Illustration).

**Central Illustration:**
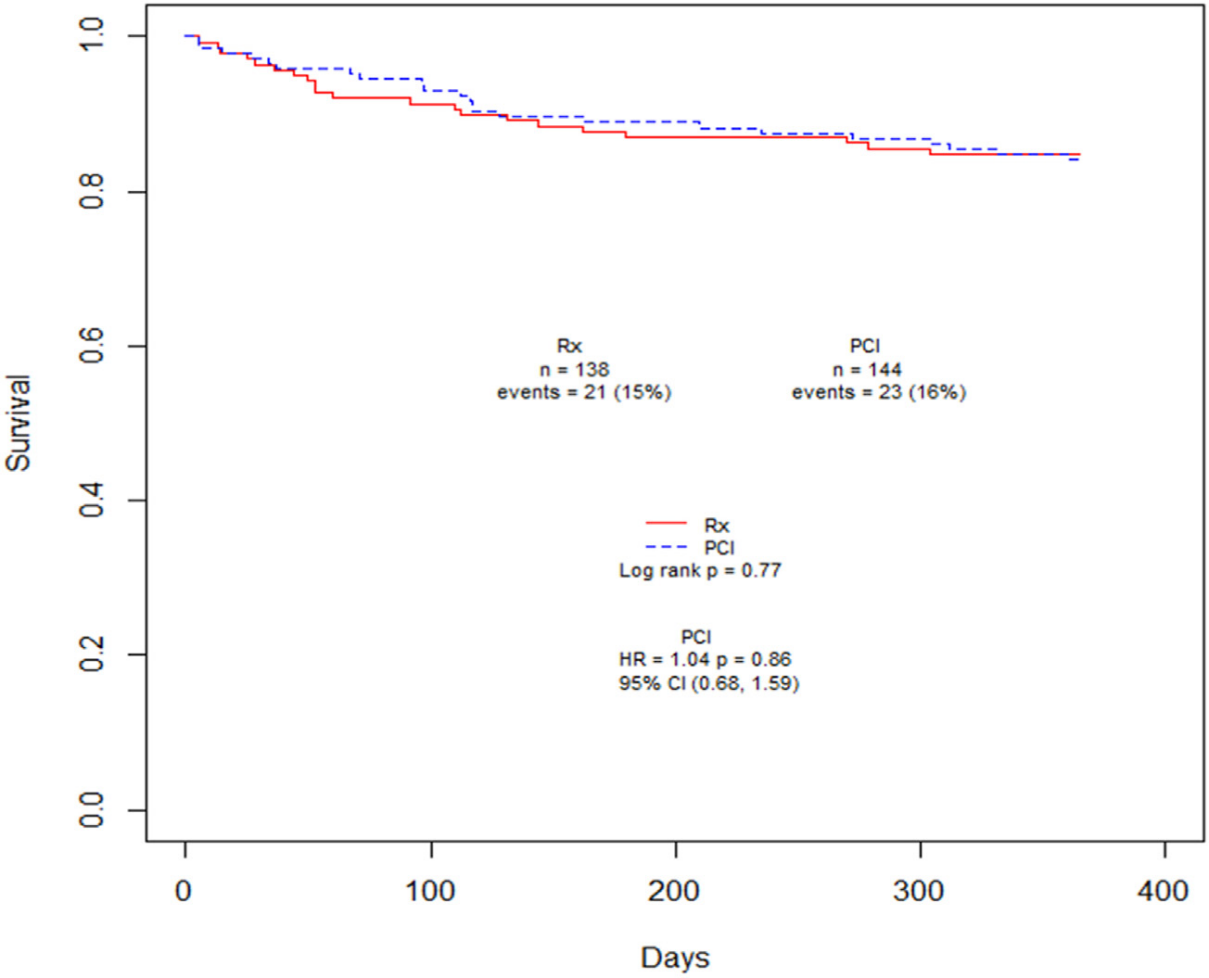
Kaplan–Meier curves showing mortality in patients with obstructive CAD undergoing TAVR as stratified by PCI or medical therapy prior to TAVR. There was no difference in mortality among patients undergoing PCI prior to TAVR vs patients who were medically managed. CAD, coronary artery disease; PCI, percutaneous coronary intervention; TAVR, transcatheter aortic valve replacement.

**Figure 4:**
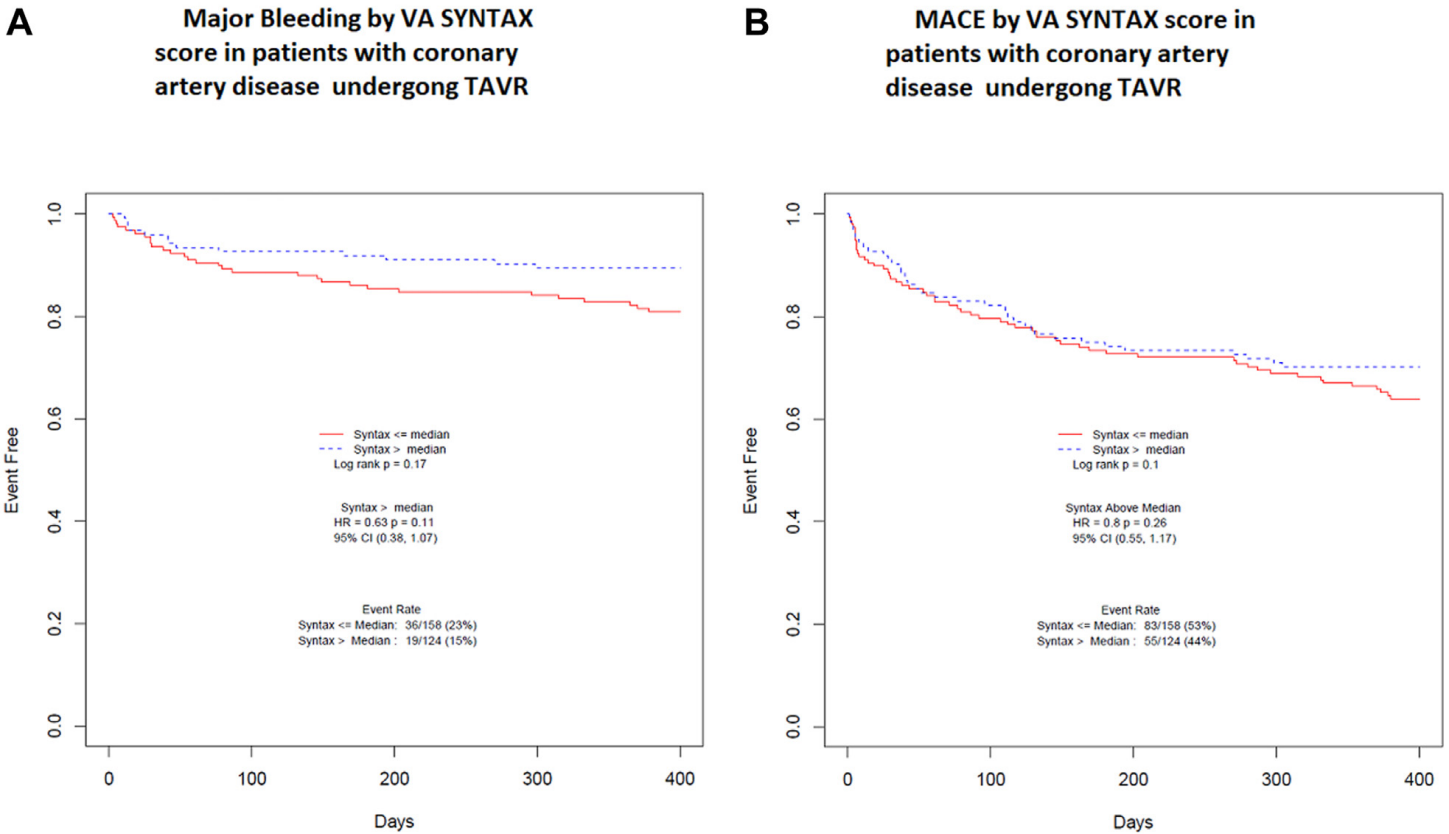
**Kaplan–Meier curves showing major bleeding** (**A**) and MACE (**B**) as stratified by VA SYNTAX score in patients with obstructive CAD undergoing TAVR. There is no difference in major bleeding or MACE in patients with high VA SYNTAX score compared with patients with low VA SYNTAX score. CAD, coronary artery disease; MACE, major adverse cardiac event; TAVR, transcatheter aortic valve replacement.

Lastly, we examined the impact of residual VA SYNTAX score on clinical outcomes. Higher residual VA SYNTAX scores, which represent an overall higher burden and complexity of unrevascularized CAD, were not associated with an increased risk of MACE at 1 year (hazard ratio [HR], 0.8; 95% CI, 0.55-1.17; *P* = .26). Higher residual VA SYNTAX scores were also not associated with increased major bleeding in patients with CAD undergoing TAVR (HR, 0.63; 95% CI, 0.38-1.07; *P* = .11) (Figure 4).

## Discussion

Although obstructive CAD is common in patients with severe symptomatic aortic stenosis, there is limited data on the ideal treatment strategy prior to aortic valve replacement. Patients with obstructive CAD enrolled in the intermediate- and low-risk PARTNER trials were more likely to have revascularization with surgery. Although a higher rate of revascularization with surgical aortic valve replacement is reasonable, the added impact of revascularization on long-term outcomes is not well understood. Moreover, as TAVR is now the dominant form of aortic valve replacement, data regarding revascularization with PCI prior to TAVR is more relevant.^8^

Contemporary data suggest increased complications with pre-TAVR PCI without meaningful gain.^9^^,^^10^ A subgroup analysis of the randomized controlled intermediate-risk SURTAVI trial also reported increased mortality with pre-TAVR PCI, but the mechanism remains unclear.^11^ Until recently, recommendations on pre-TAVR revascularization were largely based on expert opinion and data from observational studies with limited sample sizes. The ACTIVATION trial, published in 2021, is the first completed randomized controlled trial designed to assess the impact of pre-TAVR PCI on the composite end point of mortality and hospitalizations at 1 year. Although adverse event rates were similar between the 2 treatment groups, the ACTIVATION trial failed to achieve the noninferiority end point of PCI compared with medical therapy.^12^ The existing data are limited by small data sets with a heterogenous patient population; thus, extrapolation to a medically higher-risk cohort of patients is difficult.

Our study determined that the presence of obstructive CAD is an important risk modifier that renders Veteran patients at higher risk of adverse events including mortality after TAVR. We uniquely demonstrate that a significant portion of veterans have obstructive CAD prior to TAVR, rendering this population at higher overall risk for cardiovascular events. Veterans with obstructive CAD are more likely to be older and have notable comorbid conditions of heart failure and peripheral arterial disease. Overall anatomic complexity of CAD as measured by higher residual VA SYNTAX score was not associated with increased mortality, which suggests that the presence of CAD alone modified risk.

Although the presence of obstructive CAD is associated with increased mortality, there is a current gap in knowledge about how to best treat CAD, especially in a Veteran population with multiple comorbid conditions. We found that roughly half of patients with obstructive CAD underwent PCI prior to TAVR, which may be due to a lack of guideline recommendations in this space. Baseline characteristics of patients undergoing pre-TAVR PCI were similar to those that did not undergo coronary revascularization, suggesting that local practice patterns may guide the decision to revascularize rather than patient characteristics. Importantly, the percentage of pre-TAVR PCI declined over the study period, which likely represents a changing treatment paradigm over the years based on clinical experience. Our study demonstrated no difference in 1-year mortality or bleeding between those that underwent PCI and those that did not prior to TAVR.

There are several potential explanations for our findings. First, the increased mortality in patients with obstructive CAD is reasonable, as this group was also more likely to have ischemic heart failure and peripheral arterial disease. Each of these conditions likely exerts an additive effect on mortality. Second, most pre-TAVR revascularization occurs for stable or asymptomatic heart disease, in which revascularization has not been shown to reduce mortality in clinical trials.^13^ Our findings that higher residual VA SYNTAX scores were not associated with increased mortality further reiterates that revascularization may not change outcomes in stable ischemic heart disease even in the anatomically complex lesions. Third, no difference in bleeding outcome with pre-TAVR PCI may be related to dual antiplatelet therapy in all patients undergoing TAVR during the study period. It is possible that pre-TAVR PCI may be associated with bleeding complications in the current era where dual antiplatelet therapy is no longer routinely used post-TAVR.

Our study has several important limitations. First, our study shows association but cannot be used to demonstrate causality. For example, although patients with obstructive CAD demonstrated higher 1-year mortality, the exact mechanism of death cannot be elucidated and may not be related to cardiovascular events. Second, nearly 30% of patients were excluded from our analysis due to missing VA SYNTAX score, rendering our study population smaller than anticipated. Further analyses as stratified by baseline VA syntax score among patients with obstructive CAD were therefore not possible due to population size. Regardless, the results of our analysis remain significant. Third, due to limitations of our dataset, the exact timing of bleeding events in relation to PCI or TAVR cannot be further delineated. Additionally, the impact of specific antiplatelet or anticoagulants on bleeding events cannot be determined. Finally, because this is an observational study, there is a risk of unmeasured confounding.

## Conclusion

Within a large nationwide patient cohort with advanced age and multiple comorbid conditions, pre-TAVR PCI is not associated with decreased mortality advantage, which is congruent with data seen in lower-risk populations. Further research is needed to identify noninvasive strategies to reduce all-cause mortality in veterans with obstructive CAD, including optimal medical therapy and lifestyle therapies.

## References

[1] Osnabrugge R.L.J., Mylotte D., Head S.J. (2013). Aortic stenosis in the elderly: disease prevalence and number of candidates for transcatheter aortic valve replacement: a meta-analysis and modeling study. J Am Coll Cardiol.

[2] Carroll J.D., Mack M.J., Vemulapalli S. (2020). STS-ACC TVT registry of transcatheter aortic valve replacement. J Am Coll Cardiol.

[3] Kumar V., Sandhu G.S., Harper C.M., Ting H.H., Rihal C.S. (2020). Transcatheter aortic valve replacement programs: clinical outcomes and developments. J Am Heart Assoc.

[4] Stefanini G.G., Stortecky S., Wenaweser P., Windecker S. (2014). Coronary artery disease in patients undergoing TAVI: why, what, when and how to treat. EuroIntervention.

[5] Minten L., Wissels P., McCutcheon K. (2022). The effect of coronary lesion complexity and preprocedural revascularization on 5-year outcomes after TAVR. J Am Coll Cardiol Intv.

[6] Hall P.S., O’Donnell C.I., Mathew V. (2019). Outcomes of veterans undergoing TAVR within Veterans Affairs medical centers: insights from the Veterans Affairs Clinical Assessment, Reporting, and Tracking Program. J Am Coll Cardiol Intv.

[7] Valle J.A., Glorioso T.J., Bricker R. (2019). Association of coronary anatomical complexity with clinical outcomes after percutaneous or surgical revascularization in the Veterans Affairs Clinical Assessment Reporting and Tracking Program. JAMA Cardiol.

[8] Sharma T., Krishnan A.M., Lahoud R., Polomsky M., Dauerman H.L. (2022). National trends in TAVR and SAVR for patients with severe isolated aortic stenosis. J Am Coll Cardiol.

[9] Griese D.P., Reents W., Tóth A., Kerber S., Diegeler A., Babin-Ebell J. (2014). Concomitant coronary intervention is associated with poorer early and late clinical outcomes in selected elderly patients receiving transcatheter aortic valve implantation. Eur J Cardiothorac Surg.

[10] Kotronias R.A., Kwok C.S., George S. (2017). Transcatheter aortic valve implantation with or without percutaneous coronary artery revascularization strategy: a systematic review and meta-analysis. J Am Heart Assoc.

[11] Søndergaard L., Popma J.J., Reardon M.J. (2019). Comparison of a complete percutaneous versus surgical approach to aortic valve replacement and revascularization in patients at intermediate surgical risk: results from the randomized SURTAVI trial. Circulation.

[12] Patterson T., Clayton T., Dodd M. (2021). ACTIVATION (PercutAneous Coronary inTervention prIor to transcatheter aortic VAlve implantaTION): a randomized clinical trial. J Am Coll Cardiol Intv.

[13] Maron D.J., Hochman J.S., Reynolds H.R. (2020). Initial invasive or conservative strategy for stable coronary disease. N Engl J Med.

